# Mapping underweight in children using data from the five Ethiopia Demographic and Health Survey data conducted between 2000 and 2019: A geospatial analysis using the Bayesian framework

**DOI:** 10.3389/fnut.2022.988417

**Published:** 2022-09-29

**Authors:** Kendalem Asmare Atalell, Tewodros Getaneh Alemu, Chalachew Adugna Wubneh

**Affiliations:** Department of Pediatrics and Child Health Nursing, School of Nursing, College of Medicine and Health Sciences, University of Gondar, Gondar, Ethiopia

**Keywords:** Bayesian, children, geospatial analysis, mapping, underweight

## Abstract

**Background and aims:**

The Sustainable Development Goal is targeted to end all types of malnutrition including underweight by 2030. However, the reduction rate is not as expected to meet the target. Thus, we aimed to investigate the spatiotemporal distributions and drivers of underweight among children aged below 5 years in Ethiopia.

**Methods:**

Geostatistical analysis using the Bayesian framework was conducted to map the spatial and Spatiotemporal distributions of underweight. Data for the primary outcome was obtained from the Ethiopian Demographic and Health Survey 2000–2019. Covariate data were accessed from different credible online sources at high resolutions. Spatial binomial regression was fitted to identify drivers of underweight using the Bayesian approach.

**Results:**

The overall national prevalence of underweight was 44.7, 37.7, 35.4, 25.5, and 23.8% in 2000, 2005, 2011, 2016, and 2019, respectively, with a total reduction rate of 46.8%. Significant spatial clustering of underweight was observed in Northern, Northwestern, Southeastern, Eastern borders, and the border between Oromia and SNNPR regions. Mean annual temperature (mean regression coefficient (β): −0.39; 95% credible interval (95% CrI): −0.63, −0.14), altitude (β:−0.30; 95% CrI: 0.57, −0.05), population density (β:−0.03; 95% CrI: −0.03, −0.02), and distance to water bodies (β:−0.03; 95% CrI: −0.05, −0.004) were negatively associated with being underweight. However, travel time to the nearest cities in minutes (β: 0.09; 95% CrI: 0.03, 0.14) was positively associated with being underweight.

**Conclusion:**

The national prevalence of underweight is reduced slower than expected in Ethiopia, with significant spatial variations across subnational and local levels. Temperature, altitude, population density, and distance to water bodies were negatively associated with underweight, whereas travel time to the nearest cities was positively associated with underweight in Ethiopia. Improving child nutrition through creating awareness and providing clean water should be strengthened.

## Introduction

Healthy growth, appropriate organ formation, and function, as well as a strong immune system and neurological development, are all dependent on optimal nutrition for infants and early children (1). Weight for age is the WHO-recommended indicator to determine whether the child is underweight or not (2). As underweight is a composite indicator that encompasses both stunting and wasting (3). Underweight children are those whose weight for age measures is less than minus two standard deviations (−2SD) from the reference population’s median, and those who are severely underweight are those whose measures are less than minus three standard deviations (−3SD) (4).

Malnutrition and micronutrient deficiencies continue to be the most serious problems among children under the age of 5 years (5). Every year, around 5.9 million children under the age of 5 years die worldwide, with malnutrition accounting for 45% of these deaths (6). The number of people without access to adequate calories in the world has increased since 2015 (7). Despite ongoing efforts have been made, child malnutrition is a major public health issue in sub-Saharan Africa, including Ethiopia (8–10). In cognizance of this, the government of Ethiopia has initiated the Growth and Transformation Plan II, Second National Nutrition Program (NNP II), and Sekota Declaration. Nonetheless, the prevalence of undernutrition is still high (11–13). In Ethiopia, the prevalence of underweight was 24%, according to the Ethiopian Demographic and Health Survey (EDHS) of 2016 (14).

Underweight children have a poorer resistance to infections and a higher risk of dying from common childhood diseases, while those who survive are subjected to recurring illnesses and delayed growth. Such youngsters are more likely to have a lower IQ, which affects not just their academic success but also their ability to work (15, 16). The effect of child malnutrition is long-lasting and goes beyond childhood (1). Sex, residency, birth order, diarrhea, child size, mothers’ education, inadequate dietary diversity, birth interval, and unprotected source of water are all known factors that contribute to being underweight (17–19).

Malnutrition reduction progress is not fast enough to achieve internationally accepted targets, such as the Sustainable Development Goal (SDG), which targeted ending all types of malnutrition by 2030 (20). The Federal Government of Ethiopia has been working to reduce undernutrition significantly through public education and providing nutritional supplements and financial support to vulnerable families (21). However, the risk factors of undernutrition are diverse and could potentially change in space, place, and time. Understanding the cluster variation of underweight and detecting spatial heterogeneity at the subnational level over time is useful to identify gaps in the performance of child nutrition improvement programs and to come up with targeted nutritional interventions toward population the population at risk. This leads to improving the nutritional status of children and accelerates disease elimination, which finally leads to a decrease in child mortality. However, studies are limited on the spatiotemporal pattern of childhood underweight and drivers in Ethiopia. Therefore, this study aimed to map underweight in Ethiopia between 2000 and 2019 through geospatial analysis using the Bayesian approach.

## Materials and methods

### Study design and setting

A secondary analysis of the Ethiopian Demographic and Health Surveys from 2000 to 2019 was conducted to investigate the spatiotemporal distribution of underweight in Ethiopia. This study was conducted in Ethiopia, which is located in East Africa. The country has an estimated total population of 115 million in 2020 (22) and accounts for almost 1.5% of the global population, with a population density of 215 people per square kilometer. Administratively Ethiopia is divided into regions and city administrations (first level), zones (second level), districts/woredas (third level), and Kebeles (lowest level).

### Participants

Children aged below 5 years included in the five EDHSs in Ethiopia were included in this analysis. All five EDHS enumeration areas were stratified into urban and rural following the nearby population and housing censuses. The EDHS used a two-stage stratified cluster sampling. In the first stage, clusters/enumeration areas were selected using probability sampling. In the second stage, households in the selected cluster were selected using probability sampling. Data for our analysis were obtained from reproductive-age women and children aged below 5 years in each of the five EDHSs.

### Data source and variables

The outcome variable for this study was underweight, obtained from the Ethiopian Demographic and Health Surveys conducted between 2000 and 2019. Five EDHS surveys were conducted (i.e., 2000, 2005, 2011, 2016, and 2019) ever. Mothers aged 15-49 years in each selected household were interviewed, and anthropometry was taken from all children aged below 5 years in each household. Underweight was defined when the weight/age of the child is below −2 Standard Deviation (SD). Geospatial covariate data were obtained from several sources with a resolution of 1 km^2^. Climatic data such as temperature and precipitation were obtained from the WorldClim website (23). Distance to the nearest cities in minutes and distance to healthcare facilities data were obtained from the Malaria Atlas Project (MAP) (24). Population density and distance to waterbody data were retrieved from WorldPop (25) and Global Lakes and Wetlands Database (GLWD), respectively. Covariates were selected based on the potential association with the outcome variable demonstrated from previous literature and the availability of high-resolution countrywide data. The polygon shapefile for the Ethiopian administrative boundaries was obtained from the Global Administrative Areas (GADEM), a free online database. The Geographic Positioning System (GIS) data were accessed from the EDHSs. The prevalence of underweight was georeferenced and linked with area-level covariates using ArcGIS.

### Data processing and analysis

After formal registration and requesting the EDHS, data were accessed at the MEASURE DHS website. The Kids Records (KR) datasets were used for this analysis. Descriptive statistics such as the prevalence of underweight in each administrative region were calculated and presented in the table. The trends for underweight were estimated in the past two decades and presented with a graph.

### Spatial analysis

Geospatial analysis using the Bayesian approach was used to generate a spatially continuous estimate of the national prevalence of underweight mapped at 2000, 2005, 2011, 2016, and 2019 EDHS surveys at a resolution of 1 km^2^. The binomial regression model was fitted within the Bayesian framework to the prevalence of underweight of both fixed effects and geostatistical random effects. Six models were constructed separately for the prevalence of underweight in 2000–2019, 2000, 2005, 2011, 2016, and 2019 EDHS data. The model for the underweight was the same for all six datasets. A spatial binomial regression model was fitted for underweight survey data including fixed effects for temperature, precipitation, travel time to the nearest city, distance to the nearest health facilities, distance to the water body and population density, and geostatistical random effects (26). The prevalence of stunting was taken at each surveyed location *j* as the outcome variable, which was assumed to follow a binomial distribution: *Y*_*j*_ ∼ *Binomial* (*n*_*j*_,*p*_*j*_); where *Y_j_* are the observed stunted children, *n_j_* is the total number of children in each survey, and *p_j_* is the predicted prevalence of underweight at location *j* (*j* = 1, …2,547 for 2000–2019, 535 for 2000, 517 for 2005, 571 for 2011, 619 for 2016, and 305 for 2019 EDHSs). The mean predicted prevalence of underweight was modeled *via* a logit link function to a linear predictor defined as: $l\,o\,g\,i\,t\,\left(p_{j}\right)=\alpha +\sum_{z=1}^{z}\beta_{z}\,{\text{X}}_{z,j}+\zeta_{j}$; where α is the intercept, β is a matrix of covariate coefficients, ***X*** is a design matrix of *z* covariates, and ζ_*j*_ are spatial random effects modeled using a zero-mean Gaussian Markov random field (GMRF) with a Matérn covariance function. The covariance function was defined by two parameters, namely, the range ρ, which represents the distance beyond which correlation becomes negligible (approximately 0.1), and σ, which is the marginal standard deviation (27). Noninformative priors were used for α (uniform prior with bounds –∞ and ∞), and we set normal priors with mean = 0 and precision (the inverse of the variance) = 1 × 10^–4^ for each β. We used default priors for the parameters of the spatial random field (28). Parameter estimation was performed using the Integrated Nested Laplace Approximation (INLA) approach in R (R-INLA) (27, 29). Sufficient values (i.e., 150,000 samples) from each simulation run for the variables of interest were stored to ensure full characterization of the posterior distributions.

Predictions of underweight at unsampled locations were made at 1 km^2^ resolution by interpolating the spatial random effects and adding them to the sum of the products of the coefficients for the spatially variant fixed effects at each prediction location (30). The intercept was added, and the overall sum was back-transformed from the logit scale to the prevalence scale, providing prediction surfaces that show the estimated immunization coverage for all prediction locations. The covariate correlation matrix was checked, and altitude was removed due to its interaction with temperature.

The Watanabe Applicable Information Criterion (WAIC) statistic was used to select the best-fitting model.

## Result

A total of 36,193 children aged below 5 years were included in the analysis in the five EDHSs, which gives an average of 33.8% of underweighted children over the two decades. The overall national prevalence of underweight was 44.7, 37.7, 35.4, 25.5, and 23.8% in 2000, 2005, 2011, 2016, and 2019, respectively. The highest prevalence was observed in Amhara and Tigray regions, whereas a lower prevalence was observed in Addis Ababa (Table 1). Underweighted children are still high in Afar and Amhara regions.

**TABLE 1:** The national and regional prevalence of underweight among children aged below 5 years in Ethiopia between 2000 and 2019.

Regions	Prevalence of underweight					
	
	2000	2005	2011	2016	2019	2000-2019
Tigray	49.0	43.4	42.4	24.2	31.7	38.4
Afar	52.6	35.5	46.1	39.1	32.7	41.7
Amhara	52.8	49.6	41.8	29.0	27.6	41.8
Oromia	43.6	34.5	32.0	22.6	17.4	31.9
Somali	45.8	50.4	36.2	27.1	31.2	34.2
Benishangul	45.3	45.4	40.3	34.7	30.6	39.0
SNNPR	52.7	34.4	33.8	21.6	20.6	34.3
Gambela	41.5	28.6	26.4	20.1	17.5	26.7
Harari	27.5	30.7	25.0	20.3	18.3	23.8
Addis Ababa	14.8	13.8	9.6	4.5	5.3	9.3
Dire Dawa	32.3	26.6	33.6	26.5	18.1	28.4
Ethiopia	**44.7**	**37.7**	**35.4**	**25.5**	**23.8**	**33.8**

### Trend analysis

**FIGURE 1:**
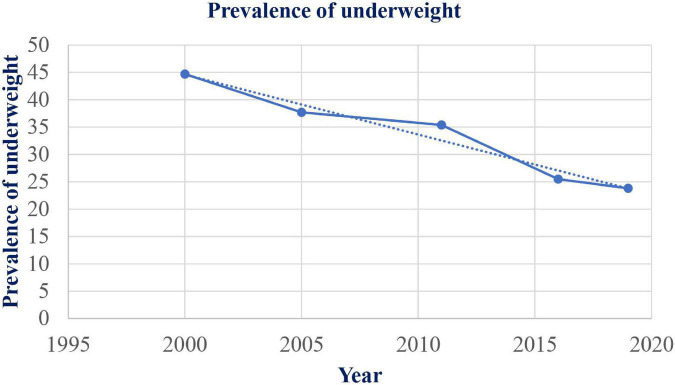
Trends of underweight among under-five children in Ethiopia between 2000 and 2019.

The prevalence of underweight was reduced from 44.7% in 2000 to 23.8% in 2019 with a total reduction rate of 46.8% (Figure 1). The reduction of underweight was faster in Addis Ababa.

### Spatial analysis

**FIGURE 2:**
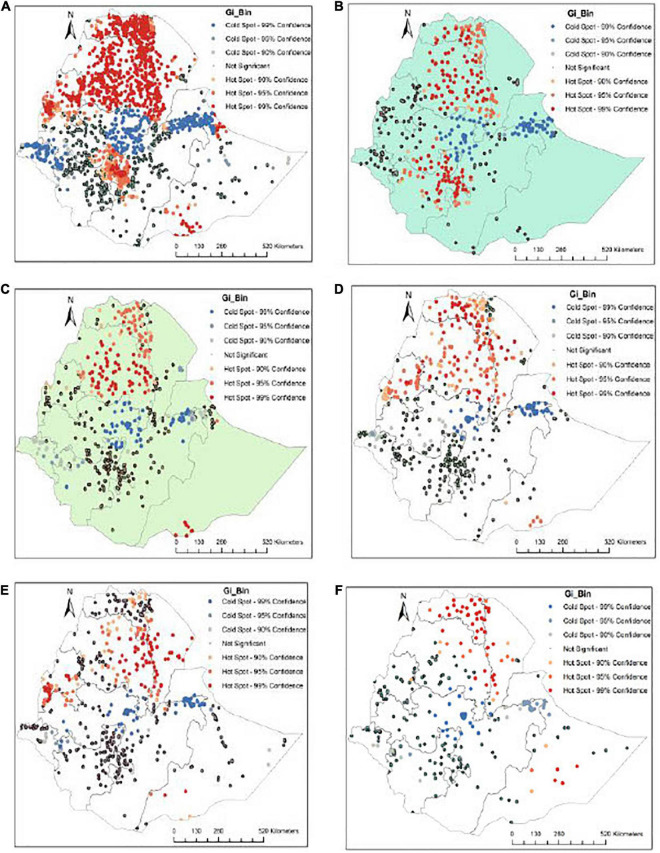
Geospatial points and prevalance of underweight in Ethiopia: **(A)** 2000-2019, **(B)** 2000, **(C)** 2005, **(D)** 2011, **(E)** 2016, and **(F)** 2019.

Significant spatial clustering of underweight was observed in Northern, Northwestern, Southeastern, Eastern borders, and the border between Oromia and SNNPR regions. However, cold spots were observed in the Western, Central, and Eastern parts of the country (Figure 2).

**FIGURE 3:**
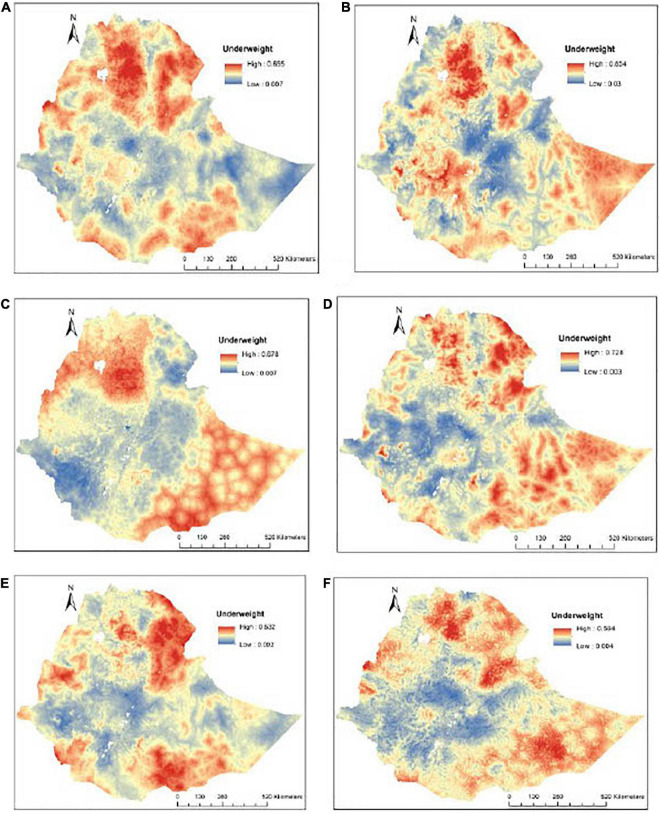
The predicted geospatial map for the prevalence of underweight in Ethiopia: **(A)** 2000-2019, **(B)** 2000, **(C)** 2005, **(D)** 2011, **(E)** 2016, and **(F)** 2019.

The predicted prevalence of underweight was observed on the Northern and Southern borders of the country (Figure 3).

### Drivers of underweight

A spatial binomial regression model was fitted using the Bayesian framework to identify drivers of underweight among children aged below 5 years in Ethiopia. Mean annual temperature (mean regression coefficient (β):−0.39; 95% credible interval (95% CrI): −0.63, −0.14), altitude (β:−0.30; 95% CrI: 0.57, −0.05), population density (β:−0.03; 95% CrI: −0.03, −0.02), and distance to water bodies (β:−0.03; 95% CrI: −0.05, −0.004) were negatively associated with being underweight. However, travel time to the nearest cities in minutes (β: 0.09; 95% CrI: 0.03, 0.14) was positively associated with being underweight (Table 2).

**TABLE 2:** Regression coefficient mean and 95% credible intervals (CrI) of covariates included in a Bayesian spatial model with Binomial response for the prevalence of underweight in Ethiopia between 2000 and 2019.

	Mean regression coefficient with 95% credible interval for each year					
	
Covariates	2000-2019	2000	2005	2011	2016	2019
Intercept	−0.55 (−0.72, −0.38)	−0.03 (−0.37, 0.28)	−0.32(−1.11, 0.60)	−0.34(−0.46, −0.21)	−0.91 (−1.15, −0.69)	-1.00 (-1.25, -0.77)
Temperature	−**0.39 (**−**0.63,**−**0.14)**	−**0.75 (**−**1.20,**−**0.30)**	0.20(−0.30,0.71)	0.14(−0.23, 0.51)	−0.27 (−0.75, 0.19)	-0.26(-1.00, 0.45)
Precipitation	0.04(−0.07, 0.16)	0.03 (−0.14, 0.20)	−0.05(−0.28, 0.19)	−0.12(−0.23, 0.00)	0.01 (−0.17, 0.20)	-0.07(-0.28, 0.16)
Altitude	−**0.30 (**−**0.57,**−**0.05)**	−0.80 (−1.28, −0.32)	0.31(−0.21, 0.84)	0.21(−0.16, 0.58)	−0.34 (−0.84, 0.15)	-0.31(-1.07, 0.39)
Travel time to cities	**0.09 (0.03, 0.14)**	**0.17 (0.06, 0.28)**	−0.04(−0.21, 0.13)	**0.19(0.09, 0.29)**	**0.15 (0.02, 0.28)**	-0.01(-0.19, 0.18)
Population density	−**0.03 (**−**0.03,**−**0.02)**	−**0.02 (**−**0.03,**−**0.01)**	−**0.03(**−**0.05,**−**0.02)**	−**0.04(**−**0.05,**−**0.03)**	−**0.04 (**−**0.05,**−**0.02)**	**-0.03(-0.04, -0.01)**
Distance to water body	−**0.03 (**−**0.05,**−**0.004)**	−0.01 (−0.06, 0.04)	0.03 (−0.05, 0.10)	0.02 (−0.03, 0.07)	−0.02 (−0.08, 0.04)	**-0.12(-0.21, -0.02)**
Distance to health facilities	0.05 (−0.03, 0.13)	0.02 (−0.21, 0.24)	0.17 (−0.09, 0.42)	0.06 (−0.09, 0.21)	0.01 (−0.17, 0.19)	0.14(-0.08, 0.35)

We used Widely Applicable Information Criteria (WAIC) statistics to identify the best-fitted model, and the model with the lowest WAIC value was the best-fitted model.

## Discussion

Undernutrition is one of the major challenges in low- and middle-income counties, particularly in Ethiopia. Underweight among children is one of the indications of poor nutrition. This study aimed to map the underweight in Ethiopia between 2000 and 2019 through geospatial analysis. The magnitude of underweight among children aged below 5 years in the past 19 years in Ethiopia was found as follows: 2000 (44.7%), 2005 (37.7%), 2011 (35.4%), 2016 (25.5%), and 2019 (23.8%), based on the EDHS data. The most recent finding from the 2019 mini-EDHS reports that 23.8% of Ethiopian children aged below 5 years were underweight.

The distribution of underweight among children aged below 5 years widely varies across the administrative region of Ethiopia. The highest proportion of underweight was reported from Amhara (41.8%) and Afar (41.7%) region followed by Benishangul 39% and Tigray 38.4% from 2000 to 2019. From these data, the lowest prevalence was observed in Addis Ababa (9.3%) of underweight children aged below 5 years. This regional disparity may be due to different agro-economical (31, 32), climate (33–35), culture (36, 37), access to information, education, healthcare service, and infrastructure (38–40). The other variation may be because Addis Ababa is the capital city of Ethiopia, which has a more urban population than the rest regions (41, 42). Urban populations have more advantages in nutrition and health-related literacy compared with the rural population disputes access to food and health service accessibility (41).

The magnitude of underweight among children has shown a significant decline from (44.7%) in 2000 to (23.8%) in 2019 in Ethiopia. This reduction trend implies that for the past 19 years in Ethiopia prevalence of underweight has decreased nearly by half with a reduction rate of 46.8%. This significant improvement in the reduction of the magnitude of underweight may be the result of the cumulative effect of different global, national, and local interventions such as improvement in the accessibility of formal education, health service, and information communication (43, 44). These conditions may avert the health and nutrition literacy of the population, which leads to changes in the behaviors of the population toward the problem (45, 46). Even though the magnitude shows a reduction, underweight is still one of the public health important problems that need national and international attention until the problem is fully addressed. In addition, the improvement is in the overall magnitude of underweight in Ethiopia generally, but the problem is still very high across the regions. Even in Addis Ababa with the lowest overall magnitude, the prevalence of underweight is still a public health concern that has to be addressed (47, 48).

In this study, the drivers of being underweight were identified. As the temperature increases, the probability of being underweight will be increased. Living in a high-temperature area have low appetite than people living in a low-temperature area, which leads to low food intake in children which contributes to being underweight. The other justification for hot (high temperature) area body fat is expected to decrease as a result of thermoregulation (49). In addition, the difference in crop production distribution between high and low temperatures may expose children to being underweight (35, 50).

The distance to the nearest cities is the other determinant of being underweight among children aged below 5 years in Ethiopia. As the distance to the nearest cities increases, the probability of being underweight will be increased. This positive association implies that those children aged below 5 years living far from cities were more vulnerable to being underweight due to the inaccessibility of health services. Those children who did not get access to a health facility may not have adequate information regarding feeding and nutrition-related information. Another possible justification is those children who did not get full access to a health facility may not be properly treated for childhood illnesses. This is one of the predisposing factors for underweight for children aged below 5 years (51, 52).

In agreement with a previous study conducted on African children (53), the population density and distance to the water body were negatively associated with being underweight in children. This is because population density is higher in cities, where nutritional literacy and healthcare access are relatively good (54). In contrast, when the distance to get clean water is far, people might take contaminated water, which leads to diarrheal diseases, which is the main cause of malnutrition in children (55).

The finding of this study implies that there is a significant improvement in the magnitude of underweight in Ethiopia between 2000 and 2019. This decline could be a result of national and global efforts to minimize the magnitude of undernutrition. The efforts may direct nutritional intervention and indirectly improve nutritional literacy. The policies and programs implemented regarding nutrition have shown remarkable success for the past 20 years in Ethiopia in the reduction of undernutrition particularly in the overall magnitude of underweight. The other important finding this study disclosed is fair and equal distribution of infrastructure including access to health facilities, especially in the remote area. In Amhara, Afar, Benishangul, and Tigray regions need special emphasis to address the very high magnitude of underweight. Health policy and programs shall follow a more innovative approach in rural areas. The strength of this study was using countrywide data that would produce reliable estimates with advanced geostatistical analysis. However, this study had some limitations, and due to the secondary nature of the data, important factors were not included due to a lack of data.

## Conclusion

The national prevalence of underweight is reduced slower than expected in Ethiopia, with significant spatial variations across subnational and local levels. Significant spatial clustering of underweight was observed in the Northern and Northeastern parts of the country. Temperature, altitude, population density, and distance to water bodies were negatively associated with underweight, whereas travel time to the nearest cities was positively associated with underweight in Ethiopia. Improving child nutrition through creating awareness and providing clean water should be strengthened.

## Data availability statement

The original contributions presented in this study are included in the article/supplementary material, further inquiries can be directed to the corresponding author.

## Author contributions

KA conceived and designed this study, run the analysis, and drafted the manuscript. TA and CW wrote the draft with KA. All authors critically reviewed the manuscript for important intellectual content and contributed to the final approval of the version to be submitted.
